# Commentary: Investigating the concept of representation in the neural and psychological sciences

**DOI:** 10.3389/fpsyg.2023.1259808

**Published:** 2023-10-27

**Authors:** Andrew Richmond

**Affiliations:** Rotman Institute, Western University, London, ON, Canada

**Keywords:** representation, conceptual reform, information, scientific concepts, cognition

## Introduction

Favela and Machery (2023) describe four experiments probing the role of the concept *representation* in the brain sciences. They show that, given short descriptions of brain activity, neuroscientists and psychologists are generally not confident whether it should be described as a representation or not. Favela and Machery interpret this to mean that the scientists are unsure what it takes for brain activity to be, or *count as*, a representation. And they conclude that the concept *representation* should either be eliminated from the brain sciences, or reformed.

The experiments are revealing, and constitute an important methodological advance on existing approaches to the concept *representation*, which mostly use a priori reflection and case studies (Ramsey, 2007; Shea, 2018; Poldrack, 2020; Baker et al., 2022). But this commentary will argue that the study's design is not well-suited to its ultimate goal, and that Favela and Machery's conclusion relies on an implausible assumption about scientific concepts. Discarding that assumption will make room for important work building on Favela and Machery's contribution.

## Scientific concepts and how to understand them

Each experiment probed “scientists' willingness to use different kinds of descriptions” (3)^1^ of brain activity, focusing on ones that describe brain activity as “representing” its environment. After seeing a “cover story about a neuroscientific study recording brain response to various stimuli” (3), participants were asked whether they agreed with a statement asserting that the brain's response represented the stimuli, responding on a 7-point Likert scale from “strongly agree” to “strongly disagree.” They were also asked about other descriptions, some involving representational notions (like “being about”) and some causal ones (like “responding to”).

Three of the four experiments modulated a particular feature of the brain activity (its scale, relation to the stimulus, and function in the brain) to probe its effect on the acceptability of representational descriptions. The fourth investigated participants' willingness to describe brain activity as *mis*representing stimuli. In each case, participants were told of the brain's response to certain stimuli, and they were asked how willing they were to describe that response as (among other things) *representational*. In other words, they were asked to categorize the brain's responses, or taxonomize them, into *representations* and *non-representations*.

For causal descriptions, like “the brain area responds to the stimulus,” responses clustered around the ends of the Likert scale. But for representational descriptions, like “the brain area represents the stimulus,” the answers clustered around the middle of the scale. The natural interpretation is that although scientists are confident in some (especially causal) categorizations of brain activity, they are not confident in their categorizations of brain activity as *representing* a stimulus or not.^2^

This is an interesting finding, and lends itself to an interesting interpretation: that scientists don't know which neural activity the concept of representation does or doesn't apply to (3). In other words, it doesn't provide a precise taxonomy of brain activity into the categories *representational* and *non-representational*. Favela and Machery conclude, on this basis, that the concept of representation must either be reformed, or simply eliminated from the brain sciences. But this conclusion does not follow from the findings, or from Favela and Machery's interpretation of them. The conclusion only follows if we make a further assumption: that what the concept *representation* contributes to science could only be a taxonomy of neural activity into the categories *representational* and *non-representational*, or that whatever it contributes must depend on that taxonomy.

This picture of a concept's scientific role looks dubious if we consider the psychology of concepts or the nuances of scientific practice. I'll summarize two reasons, before returning to the positive lessons of Favela and Machery's study. First, scientific practice shows us that taxonomy is not all scientific concepts do. When scientists conceive of misinformation as a virus, they do not assume that the concept *virus* sorts the world into two kinds of things, viruses and non-viruses, and that misinformation falls into the former category. Rather, they are using the concept to introduce modeling tools, assumptions, and conceptual frameworks to study disinformation (Kucharski, 2016). Likewise, when fluid mechanics is used to model traffic, there is no assumption that traffic *is a fluid*, or that the correct description of traffic is *as a fluid* (Sun et al., 2011). The point is to introduce modeling resources that are applicable to traffic for reasons that, while interesting, do not involve traffic's *being a fluid*. A study that presented scientists with different traffic scenarios, asking them whether they agreed with statements like “the traffic is a fluid,” would not capture the work that the concept *fluid* is doing for this area of science.

Second, there is already work that applies psychological methods to study how concepts figure into explanation; this is closely related, for obvious reasons, to questions of how concepts figure into science. Consider Lombrozo et al.s' paradigmatic work on the explanatory role of the concept *function*. Some of this work asks which things tend to be attributed functions by which populations (Lombrozo et al., 2007). But often, and more informatively, it asks what participants can *do* once they've characterized a target in terms of the concept *function*, e.g., what predictions or generalizations they can make given functional as opposed to mechanistic descriptions of a system (Lombrozo, 2009). Because the concept *function* might contribute something to explanations besides a taxonomy (of things that have functions and things that don't), this research has found ways to probe what functional descriptions are (or can be) *used to do*, rather than just what conditions elicit them.

## Discussion

Any investigation of science must confront deep philosophical questions about the structure and commitments of scientific explanation. I've focused just on one of those questions, which is central to Favela and Machery's conclusions: *what do scientific concepts contribute to the scientific project more generally?* Favela and Machery provide evidence that representational concepts in cognitive science do not provide a clear taxonomy of neural activity into the categories *representational* and *non-representational*. This is important, but, since concepts can do many things for science aside from taxonomizing its target systems, it does not support the conclusion that the concept of representation does no useful work for cognitive science. I haven't aimed to *defend* the concept of representation here. Whether it serves an important scientific role or not, whether it should be retained, reformed, or eliminated, depends on what it does for science. That can be investigated partly with a priori and case study methods (Richmond, 2023), but it must also be investigated experimentally. And if we set aside their more ambitious conclusions, Favela and Machery provide a great starting-point for that investigation.

## Author contributions

AR: Writing—original draft, Writing—review and editing.

## Footnotes

^1^All page numbers refer to Favela and Machery (2023).

^2^At least when they're asked by philosophers: a potential problem, if they're aware that philosophers tend to be very particular in how they define the concept *representation*.
